# The top 100 cited studies on bacterial persisters: A bibliometric analysis

**DOI:** 10.3389/fphar.2022.1001861

**Published:** 2022-09-13

**Authors:** Yuan Ju, Haiyue Long, Ping Zhao, Ping Xu, Luwei Sun, Yongqing Bao, Pingjing Yu, Yu Zhang

**Affiliations:** ^1^ Sichuan University Library, Sichuan University, Chengdu, China; ^2^ Department of Pharmacy, the Air Force Hospital of Western Theater Command, Chengdu, China

**Keywords:** bacterial persisters, bibliometric analysis, top-cited, citation analysis, VOSviewer

## Abstract

**Background:** Bacterial persisters are thought to be responsible for the recalcitrance and relapse of persistent infections, and they also lead to antibiotic treatment failure in clinics. In recent years, researches on bacterial persisters have attracted worldwide attention and the number of related publications is increasing. The purpose of this study was to better understand research trends on bacterial persisters by identifying and bibliometrics analyzing the top 100 cited publications in this field.

**Methods:** The Web of Science Core Collection was utilized to retrieve the highly cited publications on bacterial persisters, and these publications were cross-matched with Google Scholar and Scopus. The top 100 cited publications were identified after reviewing the full texts. The main information of each publication was extracted and analyzed using Excel, SPSS, and VOSviewer.

**Results:** The top 100 cited papers on bacterial persisters were published between 1997 and 2019. The citation frequency of each publication ranged from 147 to 1815 for the Web of Science Core Collection, 153 to 1883 for Scopus, and 207 to 2,986 for Google Scholar. Among the top 100 cited list, there were 64 original articles, 35 review articles, and 1 editorial material. These papers were published in 51 journals, and the *Journal of Bacteriology* was the most productive journal with 8 papers. A total of 14 countries made contributions to the top 100 cited publications, and 64 publications were from the United States. 15 institutions have published two or more papers and nearly 87% of them were from the United States. Kim Lewis from Northeastern University was the most influential author with 18 publications. Furthermore, keywords co-occurrence suggested that the main topics on bacterial persisters were mechanisms of persister formation or re-growth. Finally, “Microbiology” was the most frequent category in this field.

**Conclusion:** This study identified and analyzed the top 100 cited publications related to bacterial persisters. The results provided a general overview of bacterial persisters and might help researchers to better understand the classic studies, historical developments, and new findings in this field, thus providing ideas for further research.

## Introduction

Persistent bacterial infections pose significant public health problems, which increase the treatment time and costs, as well as cause death of millions of people every year (Monack et al., 2004; Chen and Wen, 2011; Martinecz and Abel zur Wiesch, 2018). Persistent bacterial infections are related to recurrent and recalcitrant infectious diseases in clinics, like implant device-related infections, lung infections of cystic fibrosis patients, urinary tract infections, and tuberculous granulomas (Singh et al., 2000; Anderson et al., 2004; Mack et al., 2004; Monack et al., 2004; Fisher et al., 2017). There are great medical challenges in treating these chronic infectious diseases as they are hard to be eradicated by antibiotics. Both the host and bacterial factors are involved in the antibiotic treatment failure, including immunosuppression, immunosurveillance, antibiotic recalcitrance, and biofilms (Monack et al., 2004; Fisher et al., 2017). Besides, the presence of bacterial persisters has attracted more attention because of their important role in persistent infections (Zhang, 2014; Fisher et al., 2017). Bacterial persisters are a small fraction of non-growing bacteria that are tolerant to antibiotics, and have been shown to accelerate the emergence of antibiotic resistance and relate to the resistance of biofilms (Cohen et al., 2013; Levin-Reisman et al., 2017; Spoering and Lewis, 2001; den Bergh et al., 2017). Bacterial persisters were first observed by Gladys Hobby (Hobby et al., 1942). She found that a small number of bacteria could survive under intensive antibiotic treatments in the culture. Then, Joseph Bigger named this subpopulation “persisters” in 1944 (Bigger, 1944). Over the last few decades, researches on bacterial persisters have made significant progress. For example, several studies showed that epigenetic factors can promote the phenotypic switching between persisters and normal-state bacteria (Balaban et al., 2004; Dhar and McKinney, 2007; Helaine et al., 2014). However, a bibliometrics analysis on bacterial persisters that reflects these advances is still lacking.

The citation frequency of a publication usually reflects its’ academic impact and the level of interest of the research community in a particular field. Therefore, a high number of citations indicate the significant contributions of this paper in the field and the potential of the paper to trigger a new research direction (Van Noorden et al., 2014; Fardi et al., 2017). Analysis of the most cited publications, especially the top 100 cited publications, provides insight into the most significant achievements of past researches over the recent decades, and this information can then be used to guide future research (Godin, 2006; Arshad et al., 2020). Currently, numerous bibliometrics studies have been performed to determine the characteristics of the most cited publications in different fields, such as emergency medicine, psychiatry, medical informatics, immunology, orthodontics, and so on. (Tsai et al., 2006; Nadri et al., 2017; Tarazona et al., 2018; Jiang et al., 2019; Zhang et al., 2019; Zhang et al., 2021). Moreover, several studies have analyzed the top 100 cited publications in tuberculosis, pneumonia, and antibiotics (Zhang et al., 2017; Wang et al., 2019; Arshad et al., 2020). However, no bibliometrics analysis has been conducted to analyze the most cited publications in the field of bacterial persisters. Hence, this study aimed to identify the top 100 cited publications on bacterial persisters, and to provide a global perspective on the current status of bacterial persisters by analyzing the publication years, number of citations, characteristics of these papers, authors, countries, institutions, keywords and subject categories.

## Materials and methods

### Search strategy and inclusion criteria

Publications concerning bacterial persisters were searched from the Web of Science Core Collection (WosCC) database on 23 June 2022. The search strategy was as follows: TS (Topics) = (persister$ OR “persistent bacteri*” OR “antibiotic persisten*” OR “antibiotic toleran*”). No restrictions on country, publication year, and language were implemented on the investigation. The search results were sorted based on the citation frequency and two authors screened the full texts to identify the top 100 cited papers on bacterial persisters. The citation counts of these papers were cross-matched with Scopus and Google Scholar. Only publications focusing on bacterial persisters were included in the bibliometric analysis, while publications that mentioned persisters in passing were excluded. Disagreements were resolved by consensus-based discussion.

### Data extraction

The following information of each publication was extracted from the WoS database: authors, title, journal, publication year, number of citations, country, institution, language, type of article, keywords, and subject categories. The impact factors (IFs) of the journals were manually supplemented from the Journal Citation Report (JCR) (Clarivate Analytics, Philadelphia, United States, available from https://jcr.clarivate.com/). For publications with two or more authors, the first-ranked author was identified as the first author, and the last-ranked author was identified as the last author. The counting of institution or country was based on the institution or country of the first author. For first authors with more than one institution or country, the first institution and country were used as country of origin. Similarly, the first category was selected for publication with more than one subject categories in the WoS database.

### Statistical analysis

Statistical analyses of descriptive data were performed using Microsoft Excel 2010 and IBM SPSS Statistic 26.0. The Spearman rank test was utilized to analyze the correlation between variables among the age of publications, citation frequency, and IFs. The *p* value < 0.05 was defined as statistically significant. The VOSviewer 1.6.17 (Leiden University, the Netherlands, available from https://www.vosviewer.com/) (van Eck and Waltman, 2010; van Eck and Waltman, 2017) was applied to map the co-occurrence network of keywords. Keywords, which appeared more than two times in the top 100 cited publications, were presented in the co-occurrence network. In the network, each circle represented a keyword and the size of the circle represented the occurrence time of the keyword. Circles in the same color represented that these keywords were included in the same cluster. The circles were connected if they appeared in the same publication and the thickness of the line represented the number of times they appeared together.

## Results

### Year of publications and citations analysis

**FIGURE 1:**
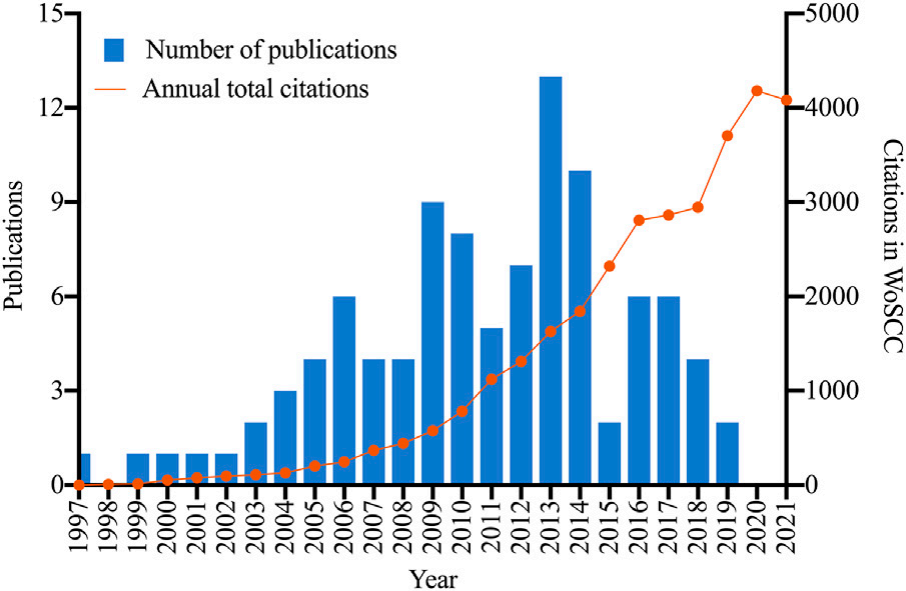
The distribution of annual publications and citations.

The top 100 cited papers were published between 1997 by Domingue and Woody (1997) and 2019 by Pang et al. (2019) and Balaban et al. (2019), but no publication in 1998 was included (Figure 1). In addition, 2013 and 2014 were the most productive years with more than 10 publications. The total citations per year of these publications showed a continuous upward trend over time, with the highest citations of 4,180 in 2020 (Figure 1).

The Top 100 cited publications on bacterial persisters along with their total citations in WoSCC, Scopus, and Google Scholar were listed in Table 1. These publications received a total of 33,638 citations in WosCC, 35,097 citations in Scopus, and 54,194 citations in Google Scholar. The citation frequency of each publication ranged from 147 to 1,815 (WoSCC), 153 to 1,883 (Scopus), and 207 to 2,986 (Google Scholar). There were only four publications that had citations more than 1,000 times in WoSCC and Scopus, but nine publications in Google Scholar. The most cited paper was published by Nathalie Q. Balaban et al. (2004) with citation frequency of 1815 (WoSCC), 1883 (Scopus), and 2,986 (Google Scholar), describing the phenotypic switch of bacterial persistence. Besides, the publication entitled “Antibiotic resistance in *Pseudomonas aeruginosa*: mechanisms and alternative therapeutic strategies” had the largest mean citations per year (*n* = 141 of WoSCC, *n* = 147 of Scopus, and *n* = 241 of Google Scholar), which was published by Zheng Pang et al.Pang et al. (2019) in Biotechnology Advances in 2019.

**TABLE 1:** The top 100 cited studies on bacterial persisters with total citation in WoSCC, Scopus, and Google Scholar databases.

				Total citations		
Rank	Title	Journal	Year	WoS^a^	ES^b^	GS^c^	IF^d^
1	Bacterial persistence as a phenotypic switch	Science	2004	1,815	1,883	2,986	31.853
2	Persister cells, dormancy and infectious disease	Nature Reviews Microbiology	2007	1,239	1,298	2,118	14.959
3	Persister Cells	Annual Review of Microbiology	2010	1,237	1,275	2,048	12.415
4	Persistence of *Mycobacterium tuberculosis* in macrophages and mice requires the glyoxylate shunt enzyme isocitrate lyase	Nature	2000	1,032	1,081	1,607	25.814
5	Platforms for antibiotic discovery	Nature Reviews Drug Discovery	2013	844	875	1,553	37.231
6	Mechanisms of antibiotic resistance in bacterial biofilms	International Journal of Medical Microbiology	2002	783	840	1,450	2.403
7	Persister cells and tolerance to antimicrobials	FEMS Microbiology Letters	2004	695	746	1,224	1.840
8	Biofilms and planktonic cells of *Pseudomonas aeruginosa* have similar resistance to killing by antimicrobials	Journal of Bacteriology	2001	618	656	1,103	3.984
9	Specialized persister cells and the mechanism of multidrug tolerance in *Escherichia coli*	Journal of Bacteriology	2004	601	634	1,021	4.146
10	Distinguishing between resistance, tolerance and persistence to antibiotic treatment	Nature Reviews Microbiology	2016	597	615	975	26.819
11	Metabolite-enabled eradication of bacterial persisters by aminoglycosides	Nature	2011	564	592	815	36.280
12	Multidrug tolerance of biofilms and persister cells	Current Topics in Microbiology and Immunology	2008	517	544	940	5.102
13	*Pseudomonas aeruginosa* Lifestyle: A Paradigm for Adaptation, Survival, and Persistence	Frontiers in Cellular and infection Microbiology	2017	508	507	836	3.520
14	Biofilm infections, their resilience to therapy and innovative treatment strategies	Journal of Internal Medicine	2012	484	503	819	6.455
15	Foamy macrophages and the progression of the human tuberculosis granuloma	Nature Immunology	2009	481	504	840	26.000
16	Ciprofloxacin Causes Persister Formation by Inducing the TisB toxin in *Escherichia coli*	Plos Biology	2010	475	509	725	12.472
17	Antibiotic tolerance facilitates the evolution of resistance	Science	2017	472	483	706	41.058
18	Silver Enhances Antibiotic Activity Against Gram-Negative Bacteria	Science Translational Medicine	2013	458	451	656	14.414
19	Recent functional insights into the role of (p)ppGpp in bacterial physiology	Nature Reviews Microbiology	2015	440	443	669	24.727
20	Internalization of Salmonella by Macrophages Induces Formation of Nonreplicating Persisters	Science	2014	427	448	626	33.611
21	Antibiotic resistance in *Pseudomonas aeruginosa*: mechanisms and alternative therapeutic strategies	Biotechnology Advances	2019	423	440	722	10.744
22	Persisters: a distinct physiological state of E-coli	BMC Microbiology	2006	421	436	698	2.896
23	Persistent bacterial infections and persister cells	Nature Reviews Microbiology	2017	418	428	657	31.851
24	Activated ClpP kills persisters and eradicates a chronic biofilm infection	Nature	2013	416	430	617	42.351
25	Molecular Mechanisms Underlying Bacterial Persisters	Cell	2014	399	420	612	32.242
26	Toxin-antitoxin systems in bacterial growth arrest and persistence	Nature Chemical Biology	2016	392	410	574	15.066
27	Mechanisms of bacterial persistence during stress and antibiotic exposure	Science	2016	386	396	579	37.205
28	Bacterial persistence: A model of survival in changing environments	Genetics	2005	384	392	650	4.289
29	The challenge of treating biofilm-associated bacterial infection	Clinical Pharmacology & therapeutics	2007	381	406	656	8.033
30	Growth Rate-Dependent Global Effects on Gene Expression in Bacteria	Cell	2009	375	400	663	31.152
31	Bacterial persistence by RNA endonucleases	Proceedings of the National Academy of Sciences of the United States of America	2011	373	397	553	9.681
32	Microbial cell individuality and the underlying sources of heterogeneity	Nature Reviews Microbiology	2006	367	385	576	15.845
33	Emergence of *Pseudomonas aeruginosa* Strains Producing High Levels of Persister Cells in Patients with Cystic Fibrosis	Journal of Bacteriology	2010	361	381	577	3.726
34	Dynamic Persistence of Antibiotic-Stressed Mycobacteria	Science	2013	342	364	515	31.477
35	Bacterial Toxin-Antitoxin Systems: More Than Selfish Entities?	Plos Genetics	2009	339	374	654	9.532
36	Bacterial Persister Cell Formation and Dormancy	Applied and Environmental Microbiology	2013	334	343	568	3.952
37	Engineered bacteriophage targeting gene networks as adjuvants for antibiotic therapy	Proceedings of the National Academy of Sciences of the United States of America	2009	324	347	542	9.432
38	Definitions and guidelines for research on antibiotic persistence	Nature Reviews Microbiology	2019	324	339	514	34.209
39	Growth of *Mycobacterium tuberculosis* biofilms containing free mycolic acids and harbouring drug-tolerant bacteria	Molecular Microbiology	2008	317	347	512	5.213
40	Persister cells and the riddle of biofilm survival	Biochemistry-Moscow	2005	311	316	650	0.858
41	Persister formation in *Staphylococcus aureus* is associated with ATP depletion	Nature Microbiology	2016	303	310	417	N/A
42	Cytological and transcript analyses reveal fat and lazy persister-like bacilli in tuberculous sputum	Plos Medicine	2008	294	229	442	12.185
43	SOS Response Induces Persistence to Fluoroquinolones in *Escherichia coli*	Plos Genetics	2009	293	313	494	9.532
44	Toxins, Targets, and Triggers: An Overview of Toxin-Antitoxin Biology	Molecular Cell	2018	292	301	419	14.548
45	Optimization of lag time underlies antibiotic tolerance in evolved bacterial populations	Nature	2014	284	296	448	41.456
46	Toxin-Antitoxin Systems Influence Biofilm and Persister Cell Formation and the General Stress Response	Applied and Environmental Microbiology	2011	284	300	435	3.829
47	Signaling-mediated bacterial persister formation	Nature Chemical Biology	2012	278	294	417	12.948
48	A Novel *In Vitro* Multiple-Stress Dormancy Model for *Mycobacterium tuberculosis* Generates a Lipid-Loaded, Drug-Tolerant, Dormant Pathogen	Plos One	2009	277	294	423	4.351
49	Characterization of the hipA7 allele of *Escherichia coli* and evidence that high persistence is governed by (p)ppGpp synthesis	Molecular Microbiology	2003	268	274	423	5.563
50	Role of persister cells in chronic infections: clinical relevance and perspectives on anti-persister therapies	Journal of Medical Microbiology	2011	268	274	432	2.502
51	Emergence of vancomycin tolerance in Streptococcus pneumoniae	Nature	1999	267	306	461	29.491
52	Structure-Activity Relationships for a Series of Quinoline-Based Compounds Active against Replicating and Nonreplicating *Mycobacterium tuberculosis*	Journal of Medicinal Chemistry	2009	265	265	324	4.802
53	Microbial Persistence and the Road to Drug Resistance	Cell Host and Microbe	2013	261	285	445	12.194
54	Characterization and Transcriptome Analysis of *Mycobacterium tuberculosis* Persisters	Mbio	2011	238	254	341	5.311
55	Regulation of phenotypic variability by a threshold-based mechanism underlies bacterial persistence	Proceedings of the National Academy of Sciences of the United States of America	2010	238	244	365	9.771
56	Molecular Mechanisms of HipA-Mediated Multidrug Tolerance and Its Neutralization by HipB	Science	2009	236	251	370	29.747
57	New antituberculosis drugs, regimens, and adjunct therapies: needs, advances, and future prospects	Lancet infectious Diseases	2014	233	254	378	22.433
58	Metabolic Control of Persister Formation in *Escherichia coli*	Molecular Cell	2013	224	235	339	14.464
59	ATP-Dependent Persister Formation in *Escherichia coli*	Mbio	2017	220	224	287	6.689
60	Microbial phenotypic heterogeneity and antibiotic tolerance	Current Opinion in Microbiology	2007	216	231	385	7.654
61	Toxin-antitoxin systems: why so many, what for?	Current Opinion in Microbiology	2010	213	223	336	7.714
62	Toxin-antitoxin modules as bacterial metabolic stress managers	Trends in Biochemical Sciences	2005	211	215	297	13.343
63	The antimicrobial peptide SAAP-148 combats drug-resistant bacteria and biofilms	Science Translational Medicine	2018	207	208	241	17.200
64	Role of global regulators and nucleotide metabolism in antibiotic tolerance in *Escherichia coli*	Antimicrobial Agents and Chemotherapy	2008	204	214	337	4.716
65	Enhanced Efflux Activity Facilitates Drug Tolerance in Dormant Bacterial Cells	Molecular Cell	2016	202	205	303	14.714
66	A new type V toxin-antitoxin system where mRNA for toxin GhoT is cleaved by antitoxin GhoS	Nature Chemical Biology	2012	199	212	318	12.948
67	Arrested Protein Synthesis Increases Persister-Like Cell Formation	Antimicrobial Agents and Chemotherapy	2013	198	203	276	4.451
68	A new class of synthetic retinoid antibiotics effective against bacterial persisters	Nature	2018	191	196	238	43.070
69	Biofilms in periprosthetic orthopedic infections	Future Microbiology	2014	187	187	294	4.275
70	Persistent bacterial infections, antibiotic tolerance, and the oxidative stress response	Virulence	2013	182	185	309	3.319
71	Phenotypic Variation of Salmonella in Host Tissues Delays Eradication by Antimicrobial Chemotherapy	Cell	2014	181	191	251	32.242
72	Toxins Hha and CspD and small RNA regulator Hfq are involved in persister cell formation through MqsR in *Escherichia coli*	Biochemical and Biophysical Research Communications	2010	168	174	253	2.595
73	A problem of persistence: still more questions than answers?	Nature Reviews Microbiology	2013	168	177	266	23.317
74	Identification of Anti-virulence Compounds That Disrupt Quorum-Sensing Regulated Acute and Persistent Pathogenicity	Plos Pathogens	2014	167	175	236	7.562
75	Eradication of bacterial persisters with antibiotic-generated hydroxyl radicals	Proceedings of the National Academy of Sciences of the United States of America	2012	166	177	247	9.737
76	Sterilizing activities of fluoroquinolones against rifampin-tolerant populations of *Mycobacterium tuberculosis*	Antimicrobial Agents and Chemotherapy	2003	165	190	300	4.246
77	Role of Oxidative Stress in Persister Tolerance	Antimicrobial Agents and Chemotherapy	2012	164	179	254	4.565
78	Increased persistence in *Escherichia coli* caused by controlled expression of toxins or other unrelated proteins	Journal of Bacteriology	2006	163	172	261	3.993
79	Formation, physiology, ecology, evolution and clinical importance of bacterial persisters	FEMS Microbiology Reviews	2017	163	168	254	11.392
80	Targeting Persisters for Tuberculosis Control	Antimicrobial Agents and Chemotherapy	2012	163	163	270	4.565
81	Ectopic overexpression of wild-type and mutant hipA genes in *Escherichia coli*: Effects on macromolecular synthesis and persister formation	Journal of Bacteriology	2006	162	166	263	3.993
82	Role of persisters and small-colony variants in antibiotic resistance of planktonic and biofilm-associated *Staphylococcus aureus*: an *in vitro* study	Journal of Medical Microbiology	2009	160	174	280	2.272
83	Phenotypic bistability in *Escherichia coli*’s central carbon metabolism	Molecular Systems Biology	2014	158	164	248	10.872
84	Starvation, Together with the SOS Response, Mediates High Biofilm-Specific Tolerance to the Fluoroquinolone Ofloxacin	Plos Genetics	2013	157	170	262	8.167
85	Multiple Toxin-Antitoxin Systems in *Mycobacterium tuberculosis*	Toxins	2014	157	155	216	2.938
86	HipA-mediated antibiotic persistence via phosphorylation of the glutamyl-tRNA-synthetase	Nature Communications	2013	156	166	229	10.742
87	*Pseudomonas aeruginosa* Increases Formation of Multidrug-Tolerant Persister Cells in Response to Quorum-Sensing Signaling Molecules	Journal of Bacteriology	2010	155	176	272	3.726
88	Evaluation of short synthetic antimicrobial peptides for treatment of drug-resistant and intracellular *Staphylococcus aureus*	Scientific Reports	2016	154	158	192	4.259
89	Isocitrate lyase mediates broad antibiotic tolerance in *Mycobacterium tuberculosis*	Nature Communications	2014	154	168	217	11.470
90	PhoU is a persistence switch involved in persister formation and tolerance to multiple antibiotics and stresses in *Escherichia coli*	Antimicrobial Agents and Chemotherapy	2007	154	157	244	4.390
91	Persistence of *Borrelia burgdorferi* in Rhesus Macaques following Antibiotic Treatment of Disseminated Infection	Plos One	2012	153	156	242	3.730
92	Persister cells, the biofilm matrix and tolerance to metal cations in biofilm and planktonic *Pseudomonas aeruginosa*	Environmental Microbiology	2005	153	162	246	4.559
93	Dormancy Is Not Necessary or Sufficient for Bacterial Persistence	Antimicrobial Agents and Chemotherapy	2013	151	155	230	4.451
94	Bridging the gap between viable but non-culturable and antibiotic persistent bacteria	Trends in Microbiology	2015	151	167	238	9.500
95	Biofilm-related disease	Expert Review of Anti-infective therapy	2018	149	155	228	3.090
96	Letting Sleeping dos Lie: Does Dormancy Play a Role in Tuberculosis?	Annual Review of Microbiology, Vol 64, 2010	2010	149	162	265	N/A
97	Carbon Sources Tune Antibiotic Susceptibility in *Pseudomonas aeruginosa* via Tricarboxylic Acid Cycle Control	Cell Chemical Biology	2017	148	156	201	5.592
98	Bacterial persistence and expression of disease	Clinical Microbiology Reviews	1997	148	146	275	8.585
99	GlpD and PlsB participate in persister cell formation in Eschetichia coli	Journal of Bacteriology	2006	147	145	237	3.993
100	Kinase activity of overexpressed HipA is required for growth arrest and multidrug tolerance in *Escherichia coli*	Journal of Bacteriology	2006	147	153	217	3.993

a WoS represented WoSCC database;

b ES represented Elsevier Scopus database.

c SC represented Google Scholar database.

d IF showed the IF of journal in the year of publication, and the N/A represented that the journal IF had not been assigned in the year of publication.

**FIGURE 2:**
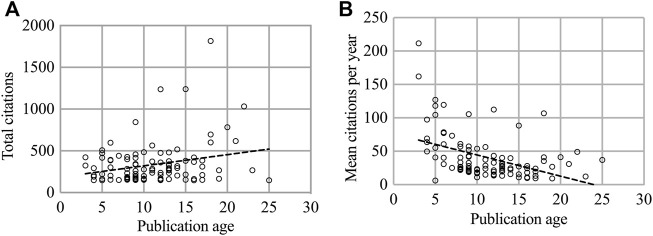
The relationship among total citations, mean citations per year, and publication age in WoSCC database. **(A)** Association of total citations with publication year (r = −0.488, *p* < 0.001 =; **(B)** Association of mean citations per year with publication age (*p* = 0.248).

The Spearman rank test indicated that there was a statistically significant downward trend in the mean citations per year with the increase in publication age (r = −0.488, *p* < 0.001 of WoSCC; r = −0.489, *p* < 0.001 of Scopus; r = −0.422, *p* < 0.005 of Google Scholar) (Figure 2A, Supplementary Figures S1A, S1B). However, no significant relationship was observed between the age of publications and the number of total citations (*p* = 0.248 of WoSCC; *p* = 0.209 of Scopus; *p* = 0.018 of Google Scholar) (Figure 2B, Supplementary Figures S1C, S1D).

### Characteristics of publications

The top 100 cited publications on bacterial persisters were all written in English. Among the publications, there were 64 original articles, 35 review articles, and one editorial material. The citation frequencies for each publication type were 20,287 (original articles), 13,183 (review articles), and 168 (editorial material). However, the type of review had the highest average citation count per paper (*n* = 377).

Overall, the top 100 cited papers on bacterial persisters were published in 51 journals (Table 2). The 2021 IFs of these journals ranged from 2.82 (*FEMS Microbiology Letters*) to 112.288 (*Nature Reviews Drug Discovery*), and 23 journals had 2021 IFs greater than 10. *Journal of Bacteriology* published the greatest number of papers (eight papers), followed by *Nature Reviews Microbiology* (seven papers) and *Antimicrobial Agents and Chemotherapy* (seven papers). In addition, *Science* had the highest total citations (3,678) with six publications. But, the highest average citation count per publication (*n* = 844) belonged to *Nature Reviews Drug Discovery*. The Spearman rank test indicated that the number of published papers (*p* = 0.255), total citations (*p* = 0.300), and average citation count per publication (*p* = 0.439) did not correlate to the 2021 IFs (Supplementary Figure S2).

**TABLE 2:** Journals of the top 100 cited publications on bacterial persisters.

Journal	Number of publications	Total citations	Average citation count per paper	2021 IF
Journal of Bacteriology	8	2,354	294	3.476
Nature Reviews Microbiology	7	3,553	508	78.297
Antimicrobial Agents and Chemotherapy	7	1,199	171	5.938
Nature	6	2,754	459	69.504
Science	6	3,678	613	63.798
Proceedings of the National Academy of Sciences	4	1,101	275	12.779
Cell	3	955	318	66.85
Molecular Cell	3	718	239	19.328
Nature Chemical Biology	3	869	289	16.29
Plos Genetics	3	789	263	6.02
Science Translational Medicine	2	665	332	19.359
Nature Communications	2	310	155	17.694
Annual Review of Microbiology	2	1,386	693	16.232
Mbio	2	458	229	7.786
Current Opinion in Microbiology	2	429	214	7.584
Applied and Environmental Microbiology	2	618	309	5.005
Molecular Microbiology	2	585	292	3.979
Plos One	2	430	215	3.752
Journal of Medical Microbiology	2	428	214	3.196
Nature Reviews Drug Discovery	1	844	844	112.288
Lancet infectious Diseases	1	233	233	71.421
Clinical Microbiology Reviews	1	148	148	50.129
Cell Host and Microbe	1	261	261	31.316
Nature Immunology	1	481	481	31.25
Nature Microbiology	1	303	303	30.964
Trends in Microbiology	1	151	151	18.23
Biotechnology Advances	1	423	423	17.681
FEMS Microbiology Reviews	1	163	163	15.177
Trends in Biochemical Sciences	1	211	211	14.264
Journal of Internal Medicine	1	484	484	13.068
Molecular Systems Biology	1	158	158	13.068
Plos Medicine	1	294	294	11.613
Plos Biology	1	475	475	9.593
Cell Chemical Biology	1	148	148	9.039
Journal of Medicinal Chemistry	1	265	265	8.039
Plos Pathogens	1	167	167	7.464
Clinical Pharmacology & therapeutics	1	381	381	6.903
Frontiers in Cellular and infection Microbiology	1	508	508	6.073
Expert Review of Anti-infective therapy	1	149	149	5.854
Environmental Microbiology	1	153	153	5.476
Virulence	1	182	182	5.428
Toxins	1	157	157	5.075
Scientific Reports	1	154	154	4.996
Current Topics in Microbiology and Immunology	1	517	517	4.737
BMC Microbiology	1	421	421	4.465
Genetics	1	384	384	4.402
International Journal of Medical Microbiology	1	783	783	3.658
Future Microbiology	1	187	187	3.553
Biochemical and Biophysical Research Communications	1	168	168	3.322
Biochemistry-Moscow	1	311	311	2.824
FEMS Microbiology Letters	1	695	695	2.82

### Countries, institutions, and authors

A total of 14 countries contributed to the top 100 cited publications on bacterial persisters (Table 3). The United States was the most productive country in this field with 64 publications. England ranked second with eight publications, followed by Israel (seven publications), Belgium (five publications), and Switzerland (three publications). Furthermore, the United States was also the most influential country on bacterial persisters with the highest total citations (23,129). New Zealand had the highest average citation count per publication (508).

**TABLE 3:** Countries of the top 100 cited publications on bacterial persisters.

Country^a^	Number of publications	Total citations	Average citation count
United States	64	23,130	361
England	8	2,676	334
Israel	7	2,239	320
Belgium	5	1,194	239
Switzerland	3	681	227
Sweden	2	924	462
Denmark	2	678	339
Canada	2	576	288
France	2	314	157
New Zealand	1	508	508
Netherlands	1	207	207
China	1	202	202
India	1	160	160
Spain	1	149	149

a The country distributions were extracted based on the country of first author.

Fifty five institutions participated in the top 100 cited publications, therein, 15 institutions have published two or more papers (Table 4). Most of these institutions were from the United States (13 institutions, 87%), and only one institution each from Israel and England. Northeastern University was the most prolific institution with 20 publications and total citations of 9,451, followed by The Hebrew University of Jerusalem (7 publications, 2,239 citations) and Boston University (5 publications, 1,885 citations). Rockefeller University ranked fourth in terms of the number of publications (3), but had the highest average citation count per publication (805).

**TABLE 4:** Institutions with more than two papers in the top 100 cited publications on bacterial persisters.

Institution^a^	Country	Number of publications	Total citations	Average citation count
Northeastern University	United States	20	9,451	472
The Hebrew University of Jerusalem	Israel	7	2,239	320
Boston University	United States	5	1,885	377
The Rockefeller University	United States	3	2,415	805
Harvard University	United States	3	464	155
University of North Dakota	United States	2	430	215
University of Illinois	United States	2	428	214
Tulane University	United States	2	301	150
Texas A&M University	United States	2	452	226
Princeton University	United States	2	375	188
Pennsylvania State University	United States	2	532	266
Newcastle University	England	2	772	386
Johns Hopkins University	United States	2	317	158
Brown University	United States	2	583	292
Broad Institute of MIT & Harvard	United States	2	348	174

a The institution distributions were extracted based on the institution of first author.

A total of 437 authors contributed to the top 100 cited publications on bacterial persisters. Among them, 13 first authors and 13 last authors published at least two papers. As shown in Table 5, Kim Lewis from Northeastern University was the most contributing author who published five first-author papers and 13 last-author papers. In terms of the first authors, Nathalie Q. Balaban from Rockefeller University and Iris Keren from Northeastern University both published three papers. It was worth noting that four first authors (Iris Keren; Brian P. Conlon; Tobias Dörr; Amy L. Spoering) were from Northeastern University, and the last author of their publications was Kim Lewis. For the last authors, James J. Collins from Boston University ranked second with six publications, followed by Kenn Gerdes from University of Copenhagen (five publications).

**TABLE 5:** Authors with more than two first-author or last-author publications in the top 100 cited publications on bacterial persisters.

First author			Last author		
Author	Institution	Number of papers	Author	Institution	Number of papers
Kim Lewis	Northeastern University	5	Kim Lewis	Northeastern University	13
Nathalie Q. Balaban	The Rockefeller University	3	James J. Collins	Boston University	6
Iris Keren	Northeastern University	3	Kenn Gerdes	University of Copenhagen	5
Brian P. Conlon	Northeastern University	2	Nathalie Q. Balaban	The Hebrew University of Jerusalem	4
Jose Luis Del Pozo	Clinical University of Navarra	2	Thomas K. Wood	Texas A&M University	4
Tobias Dörr	Northeastern University	2	John D. McKinney	The Hebrew University of Jerusalem	3
Sarah Schmidt Grant	Broad Institude of MIT & Harvard	2	Michael R. Barer	University of London	2
Alexander Harms	University of Copenhagen	2	Mark P. Brynildsen	Princeton University	2
Shaleen B. Korch	University of North Dakota	2	Thomas M. Hill	University of North Dakota	2
Etienne Maisonneuve	Newcastle University	2	Deborah T. Hung	Broad Institude MIT & Harvard	2
Amy L. Spoering	Northeastern University	2	Stanislas Leibler	The Rockefeller University	2
Laurence Van Melderena	University of Libre Bruxelles	2	Jan Michiels	Katholieke University Leuven	2
Xiaoxue Wang	Brown University	2	Marin Vulić	Northeastern University	2

### Keywords and subject categories

**FIGURE 3:**
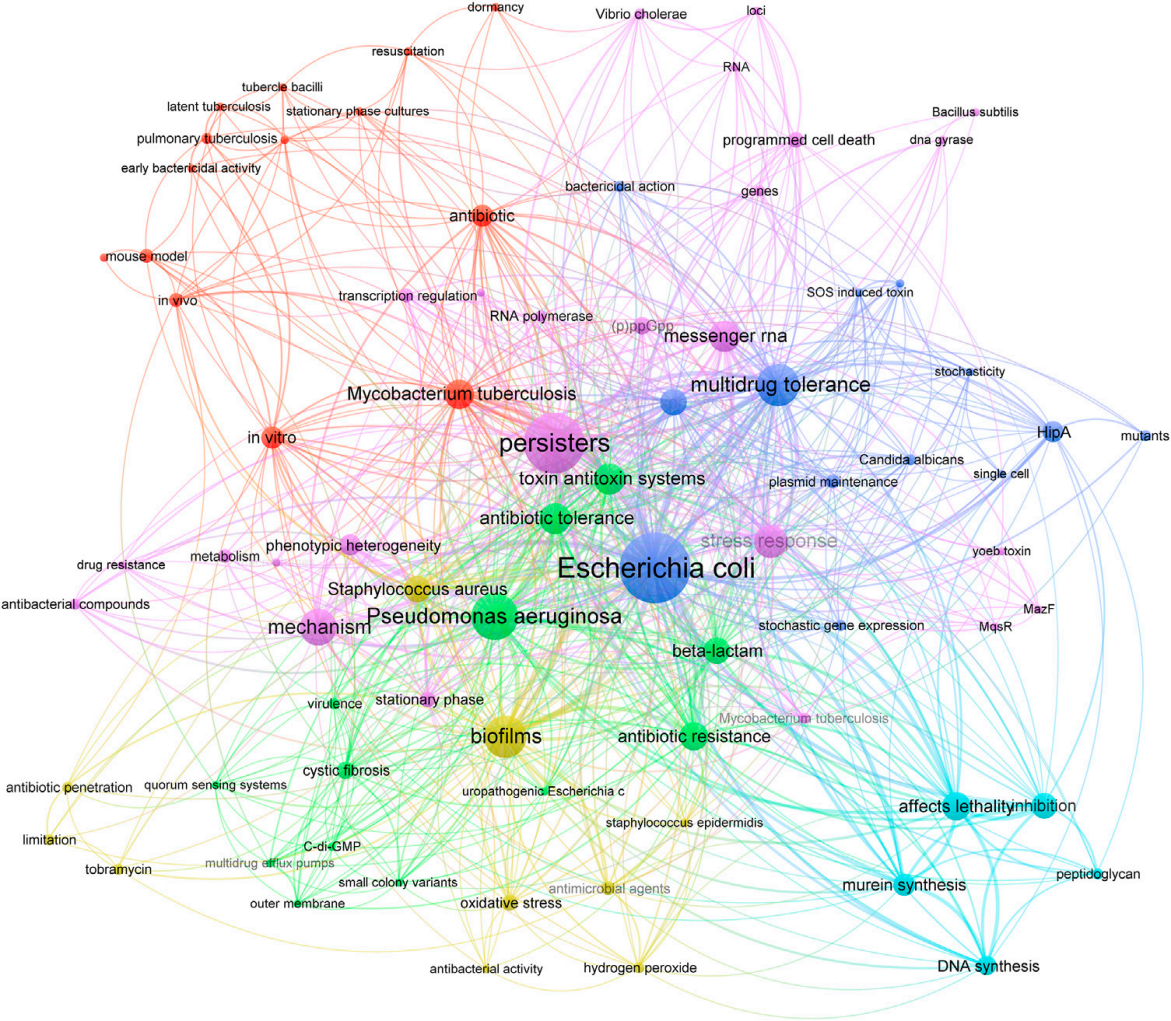
Co-occurrence network of keywords identified from top 100 cited publications on bacterial persisters. The red cluster was about the *in vitro* and *in vivo* studies on *Mycobacterium tuberculosis*. Researches on *E. coli* (blue cluster), *P. aeruginosa* (green cluster), and *S. aureus* (yellow cluster) have also been extensively studied. The yellow cluster was also focused on the role of persisters in biofilm. The purple cluster and the light blue cluster were about the mechanism of persisters formation.

Figure 3 showed the co-occurrence network of author keywords and keywords plus. The meaningless words were manually excluded and the same meaning words were merged. The most frequently occurring keywords in the top 100 cited publications were “*Escherichia coli*” (57 times), “persisters” (44 times), “*Pseudomonas aeruginosa*” (29 times), “multidrug tolerance” (25 times), “biofilms” (25 times), “mechanism” (20 times), “stress response” (17 times). Accordingly, the keywords in the network were divided into six clusters. Four clusters were separated by the research objects, including *Mycobacterium tuberculosis* (red), *E. coli* (blue), *P. aeruginosa* (green), and *Staphylococcus aureus* (yellow). From the results of co-occurrence, current researches on bacterial persisters mainly focused on 1) the role of persisters in biofilm (yellow); and 2) the mechanism of persisters formation (purple and light blue).

A total of 12 subject categories were extracted from the WoSCC. Among them, “Microbiology” was the most frequent category with 40 publications, followed by “Multidisciplinary Sciences” with 21 publications and “Biochemistry and Molecular Biology” with 18 publications. Consistent with the number of publications, the subject categories of “Microbiology” (13,088 citations) and “Multidisciplinary Sciences” (8,427 citations) had the highest total frequency of citation. Furthermore, the subject category of “Biotechnology and Applied Microbiology” had the highest average citation count per publication (*n* = 471).

## Discussion

In this study, a bibliometric study was performed to identify and analyze the top 100 cited publications on bacterial persisters. Commonly, the citation frequency of publication reflected the importance of the paper, which also indicated its scientific recognition by researchers in the relevant field, and how it generated discussion or triggered new research direction (Fardi et al., 2017; Tarazona et al., 2018). Moreover, the highly cited publication was suggested as a milestone study in the related field (Van Noorden et al., 2014). Hence, the top 100 cited publications included in this study could be identified as “classics” in the field of bacterial persisters, and the bibliometric analysis of “classics” emphasized researchers, research outputs, and future research trends (Godin, 2006).

In this study, the top 100 cited papers on bacterial persisters were published between 1997 and 2019. Among them, 2009 to 2014 was the productive period of highly cited publication on bacterial persisters (a total of 52 publications), and 2013 was found to have the most publications. The increase of publications between 2009 and 2014 indicated that more attention was paid to the field of bacterial persisters by researchers, or there were several important scientific breakthroughs during this period. The growth rate of citations increased significantly after 2009 also reflecting increasing research interest in bacterial persisters.

The citation frequency of each publication was between 147 and 1815 (WoSCC); 153 and 1883 (Scopus); 207 and 2,986 (Google Scholar). The number of citations per publication showed significant differences between databases. The research on bacterial persisters started more than 80 years ago when Hobby and Bigger found that penicillin could not fully kill culture (Hobby et al., 1942; Bigger, 1944). Therefore, the WoSCC was selected as the benchmark as it included citation metrics from 1900 to the present. The Scopus currently included citations starting in 1960, and this is a deficiency in analyzing the most cited publications (Jafarzadeh et al., 2015). Google Scholar showed higher citation frequencies as it included books, conference proceedings, dissertations, technical reports, and preprints, in addition to scientific papers (Jafarzadeh et al., 2015; Ahmad et al., 2019).

Generally speaking, earlier publications had more time to be cited and likely achieved more citations (Picknett and Davis, 1999; Jiang et al., 2019). However, our analysis discovered that the total citation counts were not related statistically to the age of publication, which was similar to the results of other bibliometric analysis (Ahmad et al., 2019; Jiang et al., 2019). The reason might be that earlier publications are possibly no longer cited as they were absorbed by new knowledge, and publications without persistent importance are more likely to be forgotten with increasing time (Garfield, 1987; Paladugu et al., 2002). Another explanation was that researchers showed more interest in the latest developments which might give in-depth research results or trigger new research directions. This viewpoint was verified by the decreasing trend in mean citation per year with age increasing.

All the top-cited papers on bacterial persisters were published in English, that’s probably because English is the most widely used language in knowledge dissemination, and several databases had a preference for English language (Seglen, 1997b; Bondi, 2017; Zhang et al., 2021). Moreover, as institutions in United States and England made significant contributions to the field of bacterial persisters, researchers from these institutions might prefer to cite papers published in English. Among the top 100 cited publications, the number of origin articles (64) was more than review articles (35). Meanwhile, the review articles had a higher average citation count per paper (*n* = 377) than origin articles (*n* = 317). Review articles generally have higher citations than other types (Seglen, 1997a). The results also reflected that review articles were important for researchers to convey their points of view, even though new findings in origin articles have gained significant attention. We found that 62 publications were included in “Microbiology” and “Multidisciplinary Sciences” subject categories, while “Biochemistry and Molecular Biology” was the focus of 18 publications. These indicated that researches on bacterial persisters involved the cross of multi-disciplinary and researchers have been working to explore the molecular mechanisms of persisters formation.

According to our bibliometric analysis, 51 different journals published the top 100 cited publications on bacterial persister. Some of the top 100 cited papers were published in journals focusing on molecular biology or pharmacy, like *Molecular Cell*, *Nature Chemical Biology*, and *Nature Reviews Drug Discovery*. This was also related to the opinion that multi-disciplinary researches have increasingly concerned with the problem of bacterial persisters. In terms of IFs, 51 papers were published in journals with 2021 IFs more than 10, and 77 papers in journals had 2021 IFs more than 5. However, the *Journal of Bacteriology*, which published the highest number of papers in the top 100 cited list, had a relatively low IF (3.476). In addition, more than half of the top papers (51) were published in journals specializing in microbiology. These facts suggested that most researchers in the field of bacterial persisters were more favored to choose reputational and authoritative journals in their research field, not only focusing on IFs. Several other factors also influenced authors to choose journals, including the difficulty of manuscript acceptance, publication charges, time of peer review and so on (Jiang et al., 2019; Shamsi et al., 2021). On the other hand, the publication cycle time and circulation time of journals also affected the citations of papers. Usually, publications in journals with a short publication cycle time and long circulation time likely gained more citation times (Jiang et al., 2019). Furthermore, journal accessibility had an impact on the citation of publications (Eysenbach, 2006).

More than half of the top 100 cited publications (64 publications) were published in the United States, while England ranked second with eight publications. In several other bibliometric studies of similar fields, the highest number of papers was also published in the United States (Arshad et al., 2020; Liu et al., 2020; Zhu et al., 2020). This reflected the great influence of the United States in the field of pathogenic bacteria. This was probably because the United States with high GDP allots more research funding to scientific investigation and some of the world’s top research institutions are located in the United States (Peng et al., 2021; Zhang et al., 2021). Besides, authors in the United States were easier to publish their studies in American journals, and they tend to cite local publications (Paladugu et al., 2002; Peng et al., 2021). Furthermore, there were 13 institutions from the United States in the list of institutions with more than two publications, while the rest two institutions were in Israel and England respectively. Among these institutions, the studies on bacterial persisters were mainly from universities, and only one research institution called Broad Institute of MIT & Harvard. Significantly, Peking University in China had one paper in the list of top 100 cited publications, and this represented the progress in the influence of our national scientific research. Previous studies have proved that China led the scientific production in several bacterial fields, however, the quality of publications from China should be further improved (Zhu et al., 2020; Quincho-Lopez and Pacheco-Mendoza, 2021).

Kim Lewis from Northeastern University was the most prolific author with the most number of publications both as first author and last author. His studies on bacterial persisters are mainly focusing on the physiological characteristics of persisters (Shah et al., 2006) and factors affecting the formation of persisters, like oxidative stress (Wu et al., 2012), ATP dependent (Conlon et al., 2016; Shan et al., 2017), antibiotics (Doerr et al., 2010), toxin/antitoxin (Correia et al., 2006; Doerr et al., 2010). Besides, Kim Lewis is also devoted to identifying novel targets or compounds to kill persisters and eradicate persistent infections (Conlon et al., 2013; Lewis, 2013). In addition, Kim Lewis cooperated with some productive authors, like Nathalie Q. Balaban from Rockefeller University, Tobias Dörr from Northeastern University, and Marin Vulić from Northeastern University.

Keywords are highly refined research content and they provide research topics and hotspots in the field. It was worth noting that keywords are not required in every publication and several papers did not display author keywords (Harms et al., 2018; Kim et al., 2018; Balaban et al., 2019). Therefore, the keywords plus from the WoSCC database were utilized to form a relatively complete network. The keywords “*Escherichia coli*”, “*Pseudomonas aeruginosa*”, “*Mycobacterium tuberculosis*” and “*Staphylococcus aureus*” occurred more than 10 times, and this indicated that researches on bacterial persisters were mainly based on studies of these strains. In addition, the high-frequency keywords belong to the mechanism of persisters formation (including “stress response,” “toxin-antitoxin systems,” “messenger-RNA,” “murein synthesis,” “HipA,” “(p)ppGpp”) and the characteristic of persisters (including “multidrug tolerance,” “biofilms,” “antibiotic resistance,” “phenotypic heterogeneity”). These keywords were regarded as the current research hotspots and future directions, which were of considerable interest to researchers around the world.

The links between different keywords were also presented in the keywords co-occurrence visualization. Regarding the mechanism studies, 27 keywords were linked to the keyword “mechanism.” In terms of total link strength, the link strengths between “mechanism” and “phenotypic heterogeneity,” “stress response,” “metabolism,” “messenger RNA,” “oxidative stress” were ranked in sequence. Phenotypic heterogeneity is generated by epigenetic regulation and it leads to the formation of nonreplicating persisters under fluctuating selective perssures (Dhar and McKinney, 2007; Helaine et al., 2014). This can be distinguished from the mechanism of bacterial resistance which is acquired by mutations or horizontal gene transfer of resistance genes (Balaban et al., 2019). The keywords “stress response,” “messenger RNA” and “oxidative stress” were also linked to “phenotypic heterogeneity.” This was probably because the phenotypic growth arrest of persisters was generated by activating stress responses. Several stress response pathways were linked to the formation of persisters, including oxidative stress, (p)ppGpp stringent response, messenger RNA involved in Toxin-antitoxin system, SOS response (Doerr et al., 2009; Vega et al., 2012; Wu et al., 2012; Maisonneuve and Gerdes, 2014; Sala et al., 2014). Keywords “inhibition,” “peptidoglycan,” “murein synthesis,” and “DNA synthesis” in the light blue cluster were related to the toxin protein HipA, which is a member of the bacterial toxin-antitoxin modules. The hipA gene regulated the establishment of a persistent state through inhibition of murein synthesis, DNA synthesis, and peptidoglycan synthesis, as well as induction of (p)ppGpp synthesis (Korch et al., 2003) (Moyed and Bertrand, 1983). The development of compounds that interfered with these pathways was an alternative strategy to kill persisters (Tkachenko et al., 2021; Kaushik et al., 2022). Furthermore, Bacterial persisters are in a state of reduced metabolism. It has been reported that carbon metabolism, nucleotide metabolism, and fatty acids metabolism were involved in the formation of bacterial persisters (McKinney et al., 2000; Hansen et al., 2008; Kotte et al., 2014). Therefore, the combination of metabolic stimuli and antibiotics (like aminoglycosides) enhanced the ability to kill persisters (Allison et al., 2011).

The role of persisters in biofilms has been extensively studied, and the keyword “biofilms” was linked to 51 (63.8%) keywords. There were strong link strengths between “biofilms” and “persisters,” this was because biofilms induce the formation of persisters, and persisters in biofilm are largely responsible for the resistance of biofilms (Lewis, 2001). Several keywords, like “toxin antitoxin system,” “stress response,” “messenger RNA,” and “oxidative stress” were linked to both “mechanism” and “biofilms.” During pathogenesis, bacteria often form a mature three-dimensional biofilm architecture through secreting extracellular matrix and cell division. In biofilms, bacteria are under the condition of nutrient limitation and therefore activing the (p)ppGpp involved stress response and triggering toxin-mediated antibiotic tolerance (Nguyen et al., 2011; Maisonneuve and Gerdes, 2014). These render the bacterial population less susceptible to antibiotics in the microenvironment conditions of biofilms, and also facilitated the formation of persisters in biofilms (Wang and Wood, 2011; Romling and Balsalobre, 2012). In addition, there were several antimicrobial agents linked to the keyword “biofilms,” such as “antibiotic,” “tobramycin,” and “beta-lactam.” Although persisters and biofilms were tolerant to antimicrobial (Keren et al., 2004), the use of antibiotics in combination with compounds to eradicate both persisters and biofilms has become a possible therapeutic strategy. For example, manifests enhanced the activity of tobramycin against *P. aeruginosa* infections (Meylan et al., 2017). However, there were relatively few keywords concerning strategies against persisters. The main reason was that the specific regulatory network for persister formation was not particularly clear. This led to few efficient targets for the development of novel inhibitors. In-depth mechanism studies were urgently needed and it continued to be the future hot spot. Furthermore, the identification of compounds with novel targets against persisters was helpful to solve the problem of chronic infection in the clinic.

To our knowledge, this study was the first bibliometric analysis to identify the top 100 cited publications on bacterial persisters. There were some limitations as with other bibliometric analyses. First, the truncated search terms “persister$”, “persisten*” and “toleran*” were utilized to define the research topics in the WoSCC database, some related publications were not included in the analysis as the diversity of the keywords. Second, although the literature search had no language restriction, it appears that only English-language studies were included in the bibliometric analysis. There were language biases in this study. Third, the top 100 cited publications were sorted by the number of total citations, while the citation frequency of publications is affected by several factors, like authors, institutions, language, and journals’ IF. In addition, earlier publications tend to achieve more citations, and some recent papers with high academic value were out of consideration as they don’t have enough time to accumulate citations. Regardless of publication year, the mean citation per year was an alternative indicator to identify the most influential publication. Moreover, self-citations have a substantial influence on the citation-based metric and this might introduce potential bias in bibliometrics. Finally, this study was a cross-sectional study and the identified top 100 cited publications could change in the future.

## Conclusion

We performed a bibliometric study of the top 100 cited publications related to bacterial persisters. These papers were published from 1997 to 2019 in 51 journals and the citation frequency of these publications presented a rising trend. The United States contributed greatly to the highly cited publications on bacterial persisters. Professor Kim Lewis from Northeastern University was the most productive author. Based on the results of keywords co-occurrence, it was found that the researches on bacterial persisters mainly focused on exploring the mechanism of persister formation or re-growth. This study provided research trends on bacterial persisters, and these might help researchers and clinical workers to better understand the classic studies and new findings in this field.

## Data Availability

The original contributions presented in the study are included in the article/Supplementary Material, further inquiries can be directed to the corresponding authors.
