# Supplementary material for: The top 100 cited studies on bacterial persisters: A bibliometric analysis
Source: Front Pharmacol. 2022 Sep 13;13:1001861. doi: 10.3389/fphar.2022.1001861 (PMC9513396; doi:10.3389/fphar.2022.1001861)
Supplement: Supplementary file 1 [file DataSheet1.docx]

Supplementary Material

##
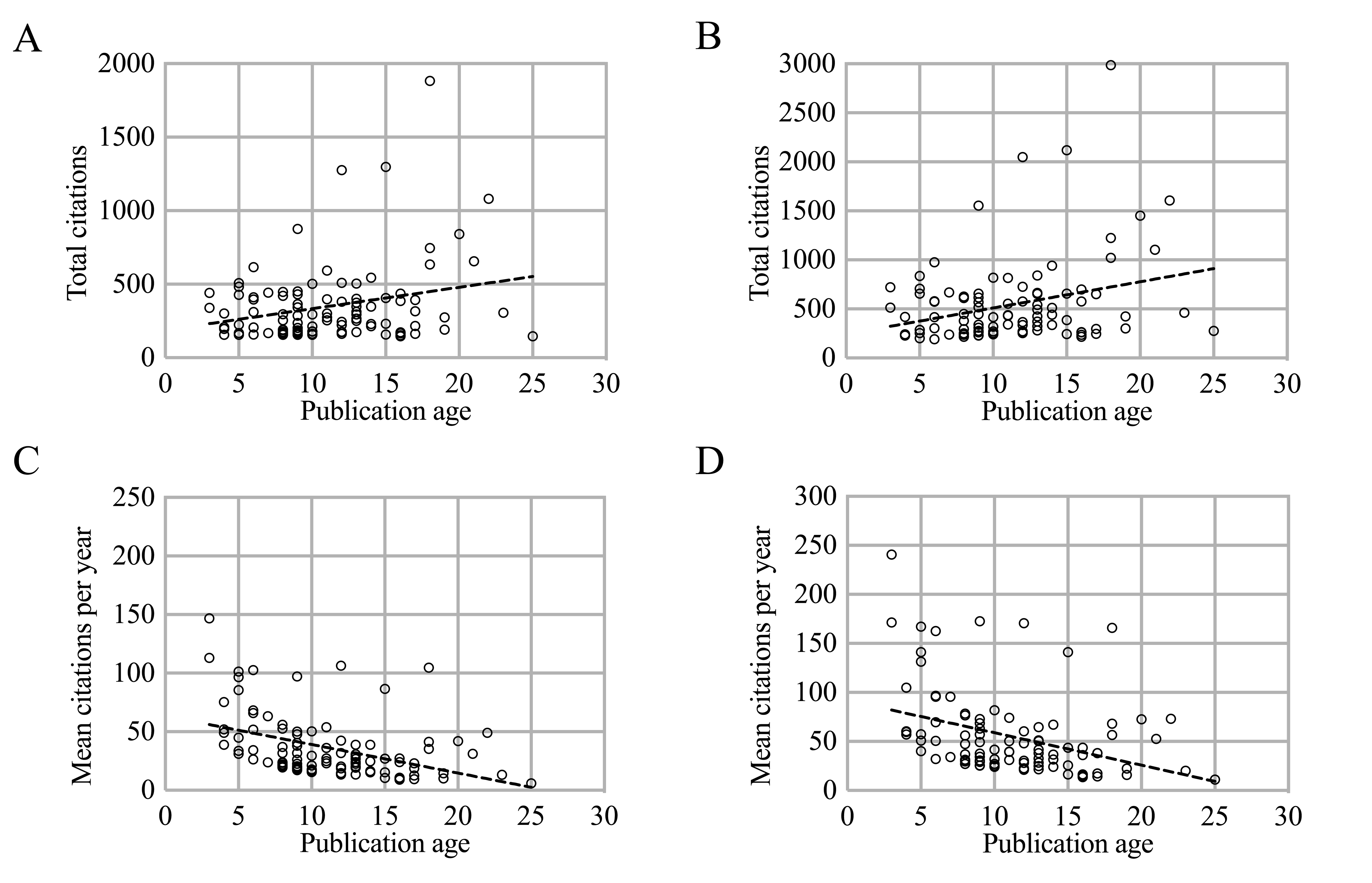


**Supplementary Figure 1**. The relationship among total citations, mean citations per year, and publication age in Scopus, and Google Scholar databases. (A) Association of total citations with publication year in Scopus database (r=-0.489, p＜0.001); (B) Association of total citations with publication year in Google Scholar database ((r=-0.422, p＜0.005); (C) Association of mean citations per year with publication age in Scopus database (*p*=0.209); (D) Association of mean citations per year with publication age in Google Scholar database (*p*=0.018).


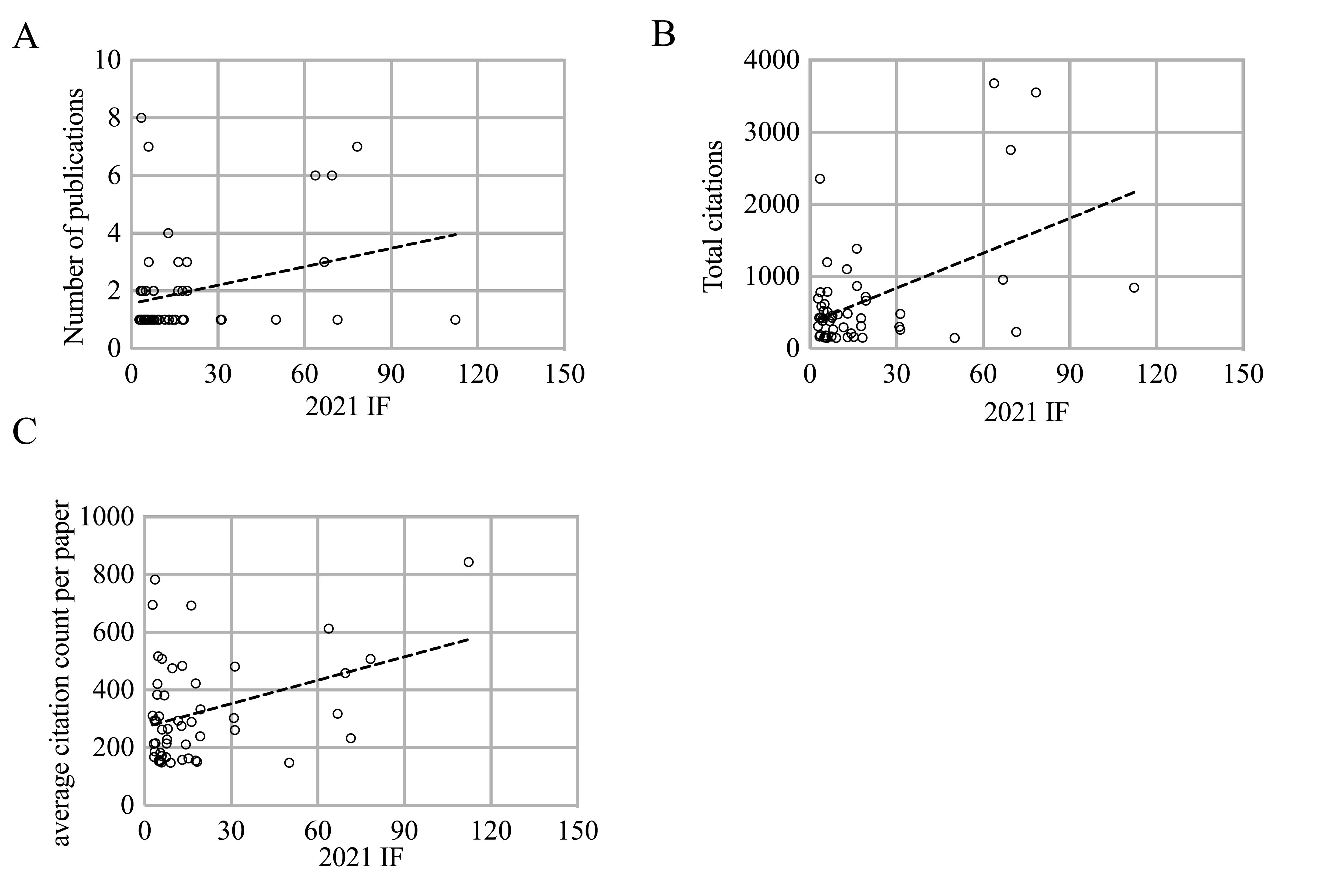


**Supplementary Figure 2**. The correlations between 2021 IF and number of publications, total citations, average citation count per paper. (A) Association of number of publications with 2021 IF (*p*=0.255); (B) Association of total citations with 2021 IF (*p*=0.300); (C) Association of average citation count per paper with 2021 IF (*p*=0.439).
